# Photo-Optical Transcutaneous Oxygen Tension Measurement Is of Added Value to Predict Diabetic Foot Ulcer Healing: An Observational Study

**DOI:** 10.3390/jcm9103291

**Published:** 2020-10-14

**Authors:** Bernard Leenstra, Robert de Kleijn, Geoffrey Kuppens, Bart Arnoldus Nicolaas Verhoeven, Jan Willem Hinnen, Gert J. de Borst

**Affiliations:** 1 Department of Vascular Surgery, UMC Utrecht, Heidelberglaan 100, 3584 CX Utrecht, The Netherlands; R.J.C.deKleijn-2@umcutrecht.nl; 2 Department of Vascular Surgery, Jeroen Bosch Ziekenhuis, Henri Dunantstraat 1, 5223 GZ ’s-Hertogenbosch, The Netherlands; geof@live.nl (G.K.); Ba.Verhoeven@jbz.nl (B.A.N.V.); j.hinnen@jbz.nl (J.W.H.)

**Keywords:** diabetic foot ulcer, TCpO2, critical limb ischemia, perfusion, peripheral arterial occlusive disease, non-invasive diagnosis

## Abstract

Currently, transcutaneous oxygen tension measurement (TCpO2) is the most favorable non-invasive test for diabetic foot ulcer (DFU) healing prognosis. Photo-optical TCpO2 is novel, less time-consuming and more practical in use compared to regular electro-chemical TCpO2. We prospectively investigated the clinical value of photo-optical TCpO2 to predict DFU healing. Patients with suspected DFU undergoing conservative treatment underwent an ankle pressure, toe-pressure and photo-optical TCpO2 test. The primary endpoint was DFU wound healing at 12 months. Based on their clinical outcome, patients were divided into a DFU healing and DFU non-healing group. Healing was defined as fully healed ulcers and non-healing as ulcers that deteriorated under conservative treatment or that required surgical amputation. Differences between groups were analyzed and an optimal TCpO2 cut-off value was determined. In total, 103 patients were included, of which 68 patients (66%) were classified as DFU healing. The remaining 35 patients (34%) had deteriorated ulcers, of which 29 (83%) eventually required surgical amputation. An optimal TCpO2 cut-off value of 43 mmHg provided a sensitivity, specificity and odds ratio of 0.78, 0.56 and 4.4, respectively. Photo-optical TCpO2 is an adequate alternative tool to validate the vascular status of the lower extremity indicating healing prognosis in patients with DFU. Therefore, we recommend that photo-optical TCpO2 can be safely coapplied in clinical practice to assist in DFU treatment strategy.

## 1. Introduction

The diabetic foot ulcer (DFU) is a devastating result of poor glycemic control, peripheral neuropathy, peripheral vascular disease and immunosuppression in patients with diabetes mellitus [1,2]. It is estimated that one in every six DFUs result in lower limb amputation, causing DFUs to account for approximately 85% of all lower limb amputations [3,4]. Moreover, half of the patients with DFU suffer from concomitant peripheral arterial disease (PAD), significantly lowering the chance of DFU healing compared to patients without PAD [5,6]. Therefore, detection of underlying PAD in patients with DFU is crucial and an essential marker for DFU healing prognosis [7]. However, diagnosing PAD in patients with diabetes mellitus is challenging as diabetes mellitus affects the micro- and macrovascular system [8]. At the moment, transcutaneous oxygen tension measurement (TCpO2) is advocated as the best available non-invasive test to predict DFU healing [7]. Current TCpO2 measurement is conducted with an electro-chemical “Clark-electrode”, of which the technical details are explained elsewhere [9]. Recently, a novel photo-optical TCpO2 device has been introduced [10]. Photo-optical TCpO2 is based on the principle of the interaction between light and matter. A photo-optical probe consists of a light-emitting-diode and a photodiode. Oxygen molecules reflect light at a specific wavelength when they absorb light and undergo a phase shift. By capturing this emitted light from the oxygen molecule, the partial oxygen pressure can be derived [11]. A practical advantage in comparison to electro-chemical TCpO2 is that preparation of the probes is less time-consuming as calibration of the photo-optical probe is not needed and photo-optical TCpO2 is possible in a handheld device, consequently making it more applicable in primary care. The added value of photo-optical TCpO2 for DFU healing in clinical practice, however, has not been established. The purpose of this study was to evaluate the added value of DFUs healing prediction with photo-optical TCpO2.

## 2. Methods

### 2.1. Study Population 

This prospective explorative study was conducted at the Jeroen Bosch Hospital, Den Bosch, The Netherlands. The study protocol was approved by the local Ethics Committee of Noord-Brabant (NW2017-32 METC Brabant). Over a two-year period (May 2016 and May 2018), all patients with suspected peripheral arterial disease and wound(s) on the lower limb referred to the vascular outward clinic were analyzed for study eligibility and were asked to provided informed consent. The inclusion criterion was a full-thickness wound distal of the ankle in patients with diabetes. Patients were excluded if they underwent revascularization after TCpO2 measurement, were not able to consequently visit the outpatient clinic for a follow-up, did not provide informed consent or had any clinical signs of sepsis. Here, sepsis is defined as a local infection with two or more clinical signs: temperature above 38 °C or below 36 °C, heart rate above 90 beats per minute, respiratory rate above 20 breaths/min, white blood cell count above 12,000 or below 4000 cu/mm. 

### 2.2. Study Procedure 

The ankle-brachial index (ABI) and toe-pressure (TP) measurement were conducted according to the standard operating protocol with visual Doppler measurement. The photo-optical TCpO2 measurement (Précise 8008 MediCap^®^, Ulrichstein, Germany) was conducted at room temperature between 20 and 22 °C and probe temperature at 44 °C; one reference TCpO2 probe was placed thorax (sub-clavicular), and one on the forefoot. Wounds, moist skin and bony or superficial venous structures were avoided. Patients were in a resting supine position during measurement. Ankle pressure (AP), TP, and TCpO2 measurements were conducted by vascular-laboratory assistants, experienced with TCpO2 measurement. The ABI was deducted. Thereafter, clinical wound assessment was carried out by a vascular surgeon and baseline characteristics were collected. The Wound, Ischemia, and foot Infection classification system (WIfI) was used to classify the status of the wound, ischemic degree and foot infection [12]. The WIfI classification system has been validated for DFU classification [13,14]. Ischemic level for the WIfI was based on either ABI or TP, irrespective of TCpO2 measurement. Treatment strategy was determined by vascular surgeons and based on standardized European guidelines [15]. The treatment strategy was determined regardless of TCpO2 values.

### 2.3. Outcome Parameters

Based on the clinical outcome during follow-up, patients were categorized into two groups: DFU healing and no DFU healing. DFU healing was defined as clinically improved ulcer healing occurring within 12 months with maximum conservative therapy, which consisted of watchful waiting, wound debridement and wound dressings. No dermo-epidermal skin grafts or hyperbaric therapy was conducted. No DFU healing was defined as clinical deterioration of the ulcer with or without amputation within 12 months. 

### 2.4. Statistical Analysis

Descriptive statistics were used to present baseline characteristics. Categorical variables were expressed as numbers and percentages. Statistical differences were determined by chi-square, or, depending on the distribution of data, independent or Welch t-test. Box plots of ABI, TP and TCpO2 for the healing and non-healing group were demonstrated with the 5th percentile. The optimal TCpO2 cut-off value for DFU healing with TCpO2 was determined by a receiver operating characteristic (ROC)-curve, defined as the highest sensitivity and specificity. Sensitivity, specificity and odds ratios were calculated. Data analysis was conducted using Statistical Package for the Social Sciences (SPSS; version 25 IBM, Armonk, NY, USA).

## 3. Results

A total of 161 patients were analyzed for study eligibility; 43 patients received revascularization without amputation, 9 patients deceased shortly after TCpO2 measurement, 3 patients were excluded due to sepsis and 3 patients were excluded due to technical problems with TCpO2 measurement. Finally, 103 patients were included for final analysis. Baseline characteristics are demonstrated in Table 1.

A significant difference was found in sex; males (*n* = 72.69%) and females (*n* = 31.30%). No further differences between the two groups were found. Improved ulcer healing occurred in 68 (66%) patients, compared to 35 (34%) patients with deteriorated ulcers. Wound healing outcome is demonstrated in Table 2.

The majority of the ulcers were present on the toes *n* = 60 (57%). Further wound classification is demonstrated in Table 3.

Vascular tests outcomes are demonstrated in Table 4.

**Figure 1:**
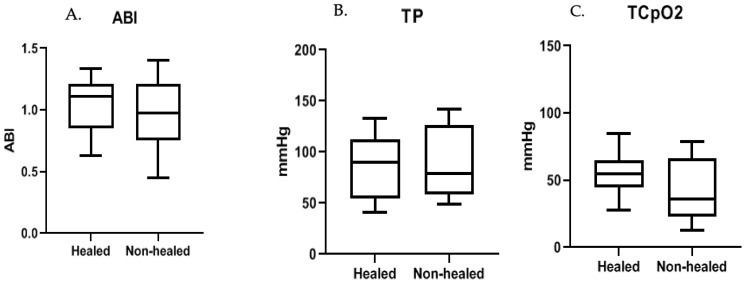
Box and whiskers ABI, TP and TCpO2. Box and whiskers of (**A**) ankle-brachial index (ABI), (**B**) toe-pressure (TP), and (**C**) transcutaneous oxygen tension measurement (TCpO2). NB. Please note different Y-axis scale between (**B**) and (**C**).

ABI, TP and TCpO2 measurements between the healing and non-healing group were 1.03 (σ 0.28) vs. 0.96 (σ 0.37) with *p* = 0.0386, 88 (σ 36) mmHg vs. 91 (σ 38) mmHg with *p* = 0.79 and 55 (σ 21) vs. 41 (σ 24) mmHg with *p* ≤ 0.05, respectively. Box and whiskers of ABI, TP and TCpO2 are demonstrated in Figure 1.

**Figure 2:**
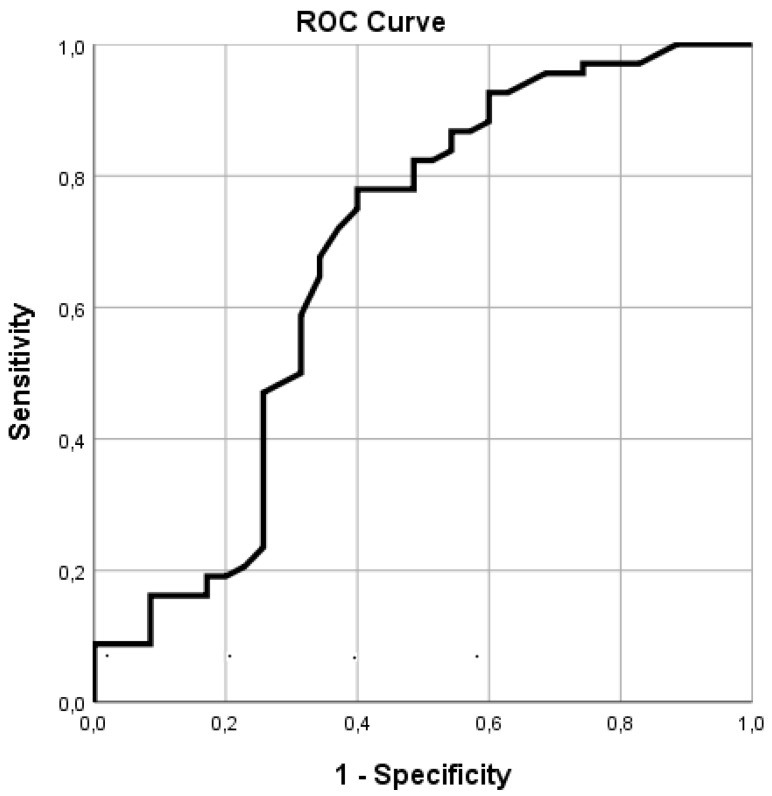
ROC-curve photo-optical TCpO2. Receiver operating characteristic (ROC)-curve of transcutaneous oxygen tension measurement (TCpO2) and wound healing.

The ROC-curve, as demonstrated in Figure 2, for TCpO2 and ulcer healing provided an Area Under the Curve of 0.67 (*p <* 0.001), providing an optimal cut-off value of 43 mmHg with a sensitivity, specificity and odds ratio of 0.78, 0.56 and 4.4, respectively.

**Figure 3:**
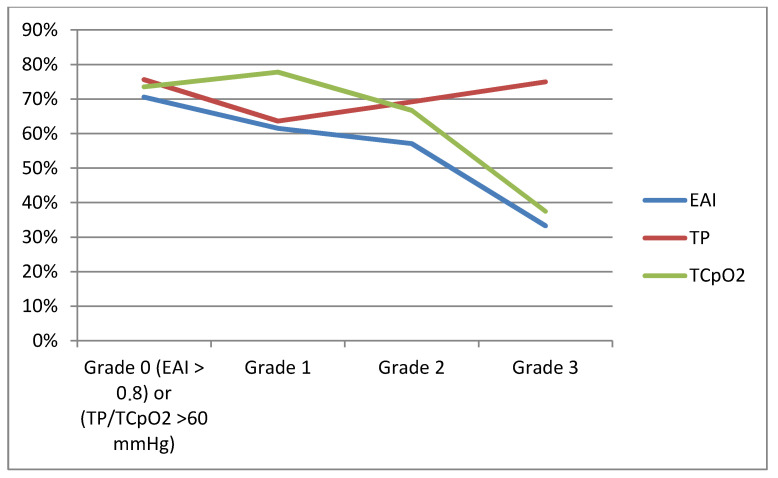
Vascular tests demonstrated within The Wound, Ischemia, and foot Infection WIfI classification grades. Ankle-Brachial Index (EAI), X-axis: Mean healing (%); Y-axis: ankle-brachial index (ABI), toe-pressure (TP) and transcutaneous oxygen tension measurement (TCpO2): Grade 0 (ABI > 0.8 or > 60 mmHg), Grade 1 (ABI 0.6–0.79 or 50–59 mmHg), Grade 2 (ABI 0.4–0.59 or 30–39 mmHg), Grade 3 (ABI < 0.39 or < 30 mmHg). Note: According to the WIfI classification grades.

The mean percentage of healing occurrence classified with the WIfI classification grade is demonstrated in Figure 3.

## 4. Discussion

In this study, we investigated the added value of the new photo-optical TCpO2 measurement for DFU healing prognosis. We found a reasonable sensitivity of 0.78 and a low specificity of 0.56 (cut-off value 43 mmHg) for TCpO2 and DFU healing prognosis. This indicates that a TCpO2 value below 43 mmHg suggests a poor chance of DFU healing. However, with an Area Under the Curve of 0.67, the diagnostic accuracy is poor to fair. Therefore, photo-optical TCpO2 cannot be used as a standalone diagnostic tool for the prognosis of DFU healing. This finding is comparable with a recent meta-analysis on electro-chemical TCpO2 and DFU healing prognosis, which found a pooled sensitivity of 0.72 [7]. This indicates that photo-optical TCpO2 is similar to the electro-chemical TCpO2 measurement in DFU healing prognosis. Two experimental studies compared photo-optical TCpO2 directly with standard electro-chemical TCpO2 and found comparative results. However, photo-optical probes tend to demonstrate higher absolute TCpO2 values [10,16]. Our findings also suggest that photo-optical probes, in contrast to electro-chemical probes, provide higher TCpO2 values. These higher values are susceptibly attributed to the fact that the photo-optical TCpO2 probes do not consume oxygen during measurement, whereas electro-chemical TCpO2 does. Therefore, some caution is advised when applying photo-optical TCpO2 values in wound classification systems [12]. Most studies and guidelines on electro-chemical TCpO2 suggest a threshold value between 25 and 30 mmHg [17,18,19,20]. In addition, minor [21] or no [18] occurrence of ulcer deterioration was found if TCpO2 was above 40 mmHg. This is not the case in our study. However, a healing probability of 73% with an electro-chemical threshold value of 40 mmHg has also been described [22]. Our study indicates that photo-optical TCpO2 demonstrates better ulcer healing occurrence in comparison to ABI or TP. This finding is in line with other studies regarding electro-chemical TCpO2 and ABI or TP [7].

Notably, in general, we found high TP values in our study population, which also explains the high percentages of DFU in Grade 3 patients shown in Figure 3. Presumably, this is attributed to the arterial stiffening caused by medial sclerosis. We found a strong concordance between high TP and high ankle pressure values. Furthermore, we found no relationship between glycated hemoglobin (HBA1c) and DFU healing impairment, although this relationship has been reported [23,24].

This study has several limitations. First, we excluded a significant number of patients with DFU who received revascularization without amputation. Naturally, revascularization is a major confounder for DFU healing and therefore these patients had to be excluded. However, if revascularization was not successful and patients afterwards had to undergo amputation due to further deterioration of the DFU, patients were included as it can be assumed that the DFU would have also showed deterioration if no revascularization had been performed. Although this provides a more homogenous study population, the decision for revascularization made by the vascular surgeons induces selection. Many factors affect the decision for revascularization, such as consent, the physical condition of the patient, interpretation of the severity of the ulcer and the degree of peripheral vascular disease. Second, a significant number of DFUs (30%) showed at least some signs of infection. It is known that infected DFUs have a lower chance of healing [25]. The inclusion of these patients alters the heterogeneity of the study population. However, to classify the clinical manifestation of infection is subjective and therefore difficult to rule out.

Nevertheless, these results do demonstrate the potential of photo-optical TCpO2 to aid in DFU healing prognosis. However, like electro-chemical TCpO2, it lacks the predictive power to singularly determine treatment strategy. Still, the implementation of photo-optical TCpO2 values in wound classification systems should be considered. Furthermore, photo-optical TCpO2 is a mobile device which can be easily transported and is less expensive in comparison to electro-chemical TCpO2. Therefore, it could be of added value in the primary care setting to indicate the necessity for referral to a specialized diabetic foot care unit. Currently, adequate referral of patients with DFU is a challenge and late referral of patients with DFU is a major risk factor for amputation [26]. More research to validate the added value of photo-optical TCpO2 for referral decisions in primary care is urgently awaited. Photo-optical TCpO2 has another practical advantage in comparison to electro-chemical TCpO2, in that it is less time-consuming. In contrast to photo-optical TCpO2, electro-chemical TCpO2 needs to be calibrated. Therefore, an electro-chemical TCpO2 measurement takes 40 min, whereas a photo-optical TCpO2 measurement on average only requires 20 min. It must be noted that in general, TCpO2 remains a sensitive measurement device. External factors such as room temperature or unnoticed probe leakage could possibly alter the outcome. Additionally, skin thickness or underlying venous or bony structures may affect measurement results.

## 5. Conclusions

Photo-optical transcutaneous oxygen tension measurement is of added value to predict diabetic foot ulcer healing. However, to determine diabetic foot ulcer healing prognosis, the application of photo-optical TCpO2 alone is insufficient. Photo-optical TCpO2 does provide objective guidance which can aid in DFU treatment strategy and therefore should be adopted in wound classification systems.

## Figures and Tables

**Table 1:** Baseline characteristics.

Baseline Characteristics	Healed (*n* = 68)	Non-Healed (*n* = 35)	*p*
Sex (male) (percentage)	44 (64.7)	28 (80.0)	0.109
Age (σ	71 (12.3)	69 (12.8)	0.701
BMI (*n*, %)			0.196
<25	37 (54.4)	21 (60.0)	
25–30	26 (3.2)	8 (22.9)	
30>	3 (4.4)	5 (14.3)	
*n*/a	2 (2.9)	1 (2.9)	
Smoking history (*n*, %)			0.641
Active	12 (21.8)	9 (31.0)	
Former smoker	22 (40.0)	12 (41.4)	
Non-smoker	18 (32.7)	6 (20.7)	
*n*/a	3 (5.5)	2 (6.9)	
Hypertension (%)	32 (47.1)	21 (60.0)	0.213
Dyslipidemia (%)	35 (51.5)	20 (58.8)	0.482
HBA1c (sd)	63.0 (15.8)	62.9 (17.5)	0.866

Overview of baseline characteristics. Standard Deviation (σ), Body mass index (BMI), not available (n/a), glycated hemoglobin (HBA1c).

**Table 2:** Wound healing outcome.

Wound Healing Outcome	(Patients, *n*)
Healed	68
Non-healed	35
Amputation	29
Deterioration without amputation	6

Wound healing outcome within 12 months of patients with diabetic foot ulcers (DFUs).

**Table 3:** Wound classification.

Wound Classification	All (*n* = 103)	Healed (*n* = 68)	Non-Healed (*n* = 35)	*p*
Wound location (*n*, %)				0.271
Toe	60 (58.3)	37 (54.5)	23 (65.7)	
Foot	43 (41.7)	31 (45.6)	12 (34.3)	
WIfI classification				
Wound (*n*, %)				0.003
1	81 (78.6)	60 (88.2)	21 (60.0)	
2	21 (20.4)	8 (11.8)	13 (37.1)	
3	1 (1.0)	0 (0)	1 (2.9)	
Ischemia (*n*, %)				0.231
0	43 (41.7)	32 (47.1)	11 (31.4)	
1	37 (35.9)	25 (36.8)	12 (34.3)	
2	8 (7.8)	4 (5.9)	4 (11.4)	
3	6 (5.8)	2 (2.9)	4 (11.4)	
Not measurable (*n*, %)	9 (8.7)	5 (7.4)	4 (11.4)	
Foot Infection (*n*, %)				0.194
0	72 (69.9)	50 (73.5)	22 (62.9)	
1	14 (13.6)	10 (14.7)	4 (11.4)	
2	17 (16.5)	8 (11.8)	9 (25.7)	

Overview of wound location and Wound, Ischemia, and foot Infection (WIfI) classification.

**Table 4:** Non-invasive vascular measurements.

Measurements	All (*n* = 103)	Healed (*n* = 68)	Non-Healed (*n* = 35)	*p*
Brachial pressure (mmHg, σ)	152	149 (24)	157 (28)	0.128
Ankle pressure (mmHg, σ)	150	152 (45)	145 (54)	0.531
Toe-pressure (mmHg, σ)	89	88 (36)	91 (38)	0.799
TCpO2 reference probe (mmHg, σ)	72	75 (29)	68 (22)	0.177
TCpO2 forefoot (mmHg, σ)	51	55 (21)	41 (24)	0.005
Ankle-brachial index (σ)	1.00	1.03 (0.28)	0.96 (0.37)	0.386

Comparison of non-invasive vascular measurements outcome between the healed and non-healed group. Standard Deviation (σ), transcutaneous oxygen tension measurement (TCpO2).

## References

[1] Morbach S., Furchert H., Groblinghoff U., Hoffmeier H., Kersten K., Klauke G.T., Klemp U., Roden T., Icks A., Haastert B. (2012). Long-term prognosis of diabetic foot patients and their limbs: Amputation and death over the course of a decade. Diabetes Care.

[2] Adler A.I., Erqou S., Lima T.A., Robinson A.H. (2010). Association between glycated haemoglobin and the risk of lower extremity amputation in patients with diabetes mellitus-review and meta-analysis. Diabetologia.

[3] Skrepnek G.H., Armstrong D.G., Mills J.L. (2014). Open bypass and endovascular procedures among diabetic foot ulcer cases in the United States from 2001 to 2010. J. Vasc. Surg..

[4] Edmonds M. (2013). Modern treatment of infection and ischaemia to reduce major amputation in the diabetic foot. Curr. Pharm. Des..

[5] Siersma V., Thorsen H., Holstein P.E., Kars M., Apelqvist J., Jude E.B., Piaggesi A., Bakker K., Edmonds M., Jirkovska A. (2013). Importance of factors determining the low health-related quality of life in people presenting with a diabetic foot ulcer: The Eurodiale study. Diabet. Med..

[6] Brownrigg J.R., Schaper N.C., Hinchliffe R.J. (2015). Diagnosis and assessment of peripheral arterial disease in the diabetic foot. Diabet. Med..

[7] Wang Z., Hasan R., Firwana B., Elraiyah T., Tsapas A., Prokop L., Mills J.L., Murad M.H. (2016). A systematic review and meta-analysis of tests to predict wound healing in diabetic foot. J. Vasc. Surg..

[8] Shi Y., Vanhoutte P.M. (2017). Macro- and microvascular endothelial dysfunction in diabetes. J. Diabetes.

[9] Vesterager P. (1977). Transcutaneous Po_2_ electrode. Scand. J. Clin. Lab. Investig. Suppl..

[10] Urban M., Fouasson-Chailloux A., Signolet I., Colas Ribas C., Feuilloy M., Abraham P. (2015). Comparison of two devices for measuring exercise transcutaneous oxygen pressures in patients with claudication. Vasa.

[11] Rumsey W.L., Vanderkooi J.M., Wilson D.F. (1988). Imaging of phosphorescence: A novel method for measuring oxygen distribution in perfused tissue. Science.

[12] Mills J.L., Conte M.S., Armstrong D.G., Pomposelli F.B., Schanzer A., Sidawy A.N., Andros G., Society for Vascular Surgery Lower Extremity Guidelines Writing Group (2014). The Society for Vascular Surgery Lower Extremity Threatened Limb Classification System: Risk stratification based on wound, ischemia, and foot infection (WIfI). J. Vasc. Surg..

[13] Mathioudakis N., Hicks C.W., Canner J.K., Sherman R.L., Hines K.F., Lum Y.W., Perler B.A., Abularrage C.J. (2017). The Society for Vascular Surgery Wound, Ischemia, and foot Infection (WIfI) classification system predicts wound healing but not major amputation in patients with diabetic foot ulcers treated in a multidisciplinary setting. J. Vasc. Surg..

[14] Hicks C.W., Canner J.K., Mathioudakis N., Sherman R., Malas M.B., Black J.H., Abularrage C.J. (2018). The Society for Vascular Surgery Wound, Ischemia, and foot Infection (WIfI) classification independently predicts wound healing in diabetic foot ulcers. J. Vasc. Surg..

[15] Aboyans V., Ricco J.B., Bartelink M.L.E.L., Bjorck M., Brodmann M., Cohnert T., Collet J.P., Czerny M., De Carlo M., Debus S. (2018). 2017 ESC Guidelines on the Diagnosis and Treatment of Peripheral Arterial Diseases, in collaboration with the European Society for Vascular Surgery (ESVS). Eur. Heart J..

[16] Leenstra B. (2019). Photo-optical transcutaneous oxygen tension measurement in patients with peripheral arterial disease. J. Cardiovasc. Surg..

[17] Ubbink D.T., Jacobs M.J., Tangelder G.J., Slaaf D.W., Reneman R.S. (1994). The usefulness of capillary microscopy, transcutaneous oximetry and laser Doppler fluxmetry in the assessment of the severity of lower limb ischaemia. Int. J. Microcirc. Clin. Exp..

[18] Kalani M., Brismar K., Fagrell B., Ostergren J., Jorneskog G. (1999). Transcutaneous oxygen tension and toe blood pressure as predictors for outcome of diabetic foot ulcers. Diabetes Care.

[19] Ballard J.L., Eke C.C., Bunt T.J., Killeen J.D. (1995). A prospective evaluation of transcutaneous oxygen measurements in the management of diabetic foot problems. J. Vasc. Surg..

[20] Norgren L., Hiatt W.R., Dormandy J.A., Nehler M.R., Harris K.A., Fowkes F.G., Group T.I.W. (2007). Inter-Society Consensus for the Management of Peripheral Arterial Disease (TASC II). J. Vasc. Surg..

[21] Faglia E., Clerici G., Caminiti M., Quarantiello A., Curci V., Morabito A. (2007). Predictive values of transcutaneous oxygen tension for above-the-ankle amputation in diabetic patients with critical limb ischemia. Eur. J. Vasc. Endovasc. Surg..

[22] Ladurner R., Kuper M., Konigsrainer I., Lob S., Wichmann D., Konigsrainer A., Coerper S., Beckert S. (2010). Predictive value of routine transcutaneous tissue oxygen tension (tcpO2) measurement for the risk of non-healing and amputation in diabetic foot ulcer patients with non-palpable pedal pulses. Med. Sci. Monit..

[23] Dhatariya K.K., Li Ping Wah-Pun Sin E., Cheng J.O.S., Li F.Y.N., Yue A.W.Y., Gooday C., Nunney I. (2018). The impact of glycaemic variability on wound healing in the diabetic foot-A retrospective study of new ulcers presenting to a specialist multidisciplinary foot clinic. Diabetes Res. Clin. Pract..

[24] Diabetes Control and Complications Trial Research Group (1994). Effect of intensive diabetes treatment on the development and progression of long-term complications in adolescents with insulin-dependent diabetes mellitus: Diabetes Control and Complications Trial. J. Pediatr..

[25] Lipsky B.A., Berendt A.R., Cornia P.B., Pile J.C., Peters E.J., Armstrong D.G., Deery H.G., Embil J.M., Joseph W.S., Karchmer A.W. (2013). 2012 infectious diseases society of america clinical practice guideline for the diagnosis and treatment of diabetic foot infections. J. Am. Podiatr. Med. Assoc..

[26] Manu C., Lacopi E., Bouillet B., Vouillarmet J., Ahluwalia R., Ludemann C., Garcia-Klepzig J.L., Meloni M., De Buruaga V.R., Sanchez-Rios J.P. (2018). Delayed referral of patients with diabetic foot ulcers across Europe: Patterns between primary care and specialised units. J. Wound Care.

